# Distinct Embryonic Origin and Injury Response of Resident Stem Cells in Craniofacial Muscles

**DOI:** 10.3389/fphys.2021.690248

**Published:** 2021-07-01

**Authors:** Xu Cheng, Bing Shi, Jingtao Li

**Affiliations:** State Key Laboratory of Oral Diseases, Department of Oral and Maxillofacial Surgery, National Clinical Research Center for Oral Diseases, West China Hospital of Stomatology, Sichuan University, Chengdu, China

**Keywords:** myogenesis, satellite cell, fibro-adipogenic progenitor, stem cell niche, craniofacial repair

## Abstract

Craniofacial muscles emerge as a developmental novelty during the evolution from invertebrates to vertebrates, facilitating diversified modes of predation, feeding and communication. In contrast to the well-studied limb muscles, knowledge about craniofacial muscle stem cell biology has only recently starts to be gathered. Craniofacial muscles are distinct from their counterparts in other regions in terms of both their embryonic origin and their injury response. Compared with somite-derived limb muscles, pharyngeal arch-derived craniofacial muscles demonstrate delayed myofiber reconstitution and prolonged fibrosis during repair. The regeneration of muscle is orchestrated by a blended source of stem/progenitor cells, including myogenic muscle satellite cells (MuSCs), mesenchymal fibro-adipogenic progenitors (FAPs) and other interstitial progenitors. Limb muscles host MuSCs of the *Pax3* lineage, and FAPs from the mesoderm, while craniofacial muscles have MuSCs of the *Mesp1* lineage and FAPs from the ectoderm-derived neural crest. Both *in vivo* and *in vitro* data revealed distinct patterns of proliferation and differentiation in these craniofacial muscle stem/progenitor cells. Additionally, the proportion of cells of different embryonic origins changes throughout postnatal development in the craniofacial muscles, creating a more dynamic niche environment than in other muscles. In-depth comparative studies of the stem cell biology of craniofacial and limb muscles might inspire the development of novel therapeutics to improve the management of myopathic diseases. Based on the most up-to-date literature, we delineated the pivotal cell populations regulating craniofacial muscle repair and identified clues that might elucidate the distinct embryonic origin and injury response in craniofacial muscle cells.

## Introduction

The emergence of craniofacial muscles, together with the skull and sensory organs derived from the placodes and neural crest, contributed to the development of the vertebrate head, an evolutionary novelty (Gans and Northcutt, 1983; Glenn Northcutt, 2005). The muscularized pharynx and enhanced sensory abilities facilitated a more active predatory lifestyle during the transition from invertebrates to the early vertebrates (Vyas et al., 2020). In humans, the craniofacial muscles are small-to-medium-sized skeletal muscles, of which there are approximately 70, and they are involved in physiological activities such as facial expressions, food intake, respiration, and speech (Schubert et al., 2019). These muscle functions could be easily impaired by congenital deformities, tumor invasion, and traumatic injury (desJardins-Park et al., 2019). Once affected, craniofacial muscles can only be incompletely repaired with limited treatment modalities, resulting in inadequate muscle restoration and excessive fibrotic tissue production (Schreurs et al., 2020). A lack of knowledge about craniofacial muscle regeneration might contribute to the limited treatment options (Von den Hoff et al., 2019; Schreurs et al., 2020). Because skeletal muscle possesses high regenerative capacity, in that a single transplanted myofiber can generate more than one hundred new myofibers (Collins et al., 2005), therapeutic strategies to boost craniofacial regeneration could be useful.

Unlike the extensively studied limb and trunk muscles, the characteristics of craniofacial muscles have only been investigated in recent years. Researchers have gradually realized that craniofacial muscles have disparate evolutionary origins and undergo distinct developmental trajectories from limb and trunk muscles (Diogo et al., 2015; Schubert et al., 2019; Vyas et al., 2020). Moreover, their specificity in embryonic origin is accompanied by distinct muscle regeneration processes in which the muscle stem cell behavior also differs. Upon injury, the masseter muscle is likely to undergo inadequate muscle regeneration and excessive fibrosis (Ono et al., 2010; Randolph et al., 2015). Nevertheless, the underlying mechanism remains largely unknown.

Skeletal muscle regeneration is orchestrated by the interactions among multiple resident muscle cells (Wosczyna and Rando, 2018). Of these cells, myogenic muscle satellite cells (MuSCs) and mesenchymal fibroadipogenic progenitors (FAPs) have been found to be important. MuSCs play a principal role in the initiation and completion of myogenesis in response to acute or chronic injury (Dumont et al., 2015a; Wosczyna and Rando, 2018). They proliferate and differentiate to produce myogenic progenitor cells, which ultimately fuse with each other to reconstitute myofiber integrity and function (Dumont et al., 2015b). Meanwhile, FAPs have a vital function in extracellular matrix deposition, which is equally indispensable to skeletal muscle regeneration (Bentzinger et al., 2012; Yin et al., 2013; Biferali et al., 2019). Accumulating evidence indicates the distinct cellular behavior of MuSCs and FAPs in the craniofacial region. Understanding the differential regulatory mechanisms involved in muscle regeneration in the craniofacial region and other anatomic regions would support the development of novel muscle repair strategies.

In this review, we aim to provide an up-to-date discussion of the unique properties of the major cell groups in craniofacial muscles, ranging from their embryonic origin to injury response.

## The Architect of Muscle Regeneration: Satellite Cells

Muscle satellite cells are peripherally located myofiber-associated stem cells wedged between the plasma membrane and basal lamina (Dumont et al., 2015a). MuSCs have a large nuclear-to-cytoplasmic ratio, which is a morphological feature typical of stem cells (Dumont et al., 2015a). The MuSC population is characterized by the expression of the transcription factor *Pax7* (Dumont et al., 2015b) and can be isolated by flow cytometry via the surface markers Intergrin-α7 and Vcam1 (Dumont et al., 2015b). In healthy, unstressed muscle, MuSCs are mitotically quiescent (Baghdadi et al., 2018). When activated, MuSCs undergo either symmetric or asymmetric division (Forbes and Rosenthal, 2014). Symmetric division enables MuSCs to replenish the stem cell pool, while asymmetric division facilitates myogenic differentiation and MuSC self-renewal (Feige et al., 2018).

### Craniofacial MuSCs Are of Diverse Embryonic Origins

Both the craniofacial muscles and the trunk/limb muscles derive from the mesoderm (Diogo et al., 2015; Cao et al., 2019; Schubert et al., 2019), but there are many embryonic differences. The craniofacial muscles are derivatives of the cranial mesoderm, while the trunk/limb muscles are derivatives of the lateral plate mesoderm, leading to distinct developmental patterns and genetic lineages in the two muscle groups (Vyas et al., 2020). In addition, there is substantial heterogeneity in developmental origins within the craniofacial muscles among the subgroups (Lescroart et al., 2010).

In the established model of trunk/limb muscle development, the lateral plate mesoderm is segmented into somites under local oscillations of gene expression (Chang and Kioussi, 2018; Vyas et al., 2020). The somites of the *Pax3/Pax7* lineage then give rise to the corresponding trunk/limb muscles in different body segments (Harel et al., 2009). In contrast, the cranial mesoderm generates three discrete developmental units based on their general function and developmental origin (Schubert et al., 2019; Sefton and Kardon, 2019; Vyas et al., 2020): (1) *Mesp1/Pitx2* lineage extraocular muscles (EOM) derived from the prechordal mesoderm (Harel et al., 2009; Keefe et al., 2015); (2) *Mesp1/Isl1* lineage pharynx/cranial openings-associated muscles derived from pharyngeal arches (Harel et al., 2009; Schubert et al., 2019); (3) *Mesp1/Pax3* lineage tongue muscles derived from the most anterior somites (Harel et al., 2009; Diogo et al., 2015; Heude et al., 2018). Thus, the organization of the cranial mesoderm is more precisely delineated by molecular markers than by anatomical boundaries. In particular, the *Mesp1/Isl1* lineage craniofacial muscles and the cardiac muscle share the same developmental origin: the cardiopharyngeal field (Diogo et al., 2015). The critical cardiac lineage marker *Isl1* also mediates craniofacial muscle myogenesis (Harel et al., 2009; Chan et al., 2013; Vyas et al., 2020).

**FIGURE 1:**
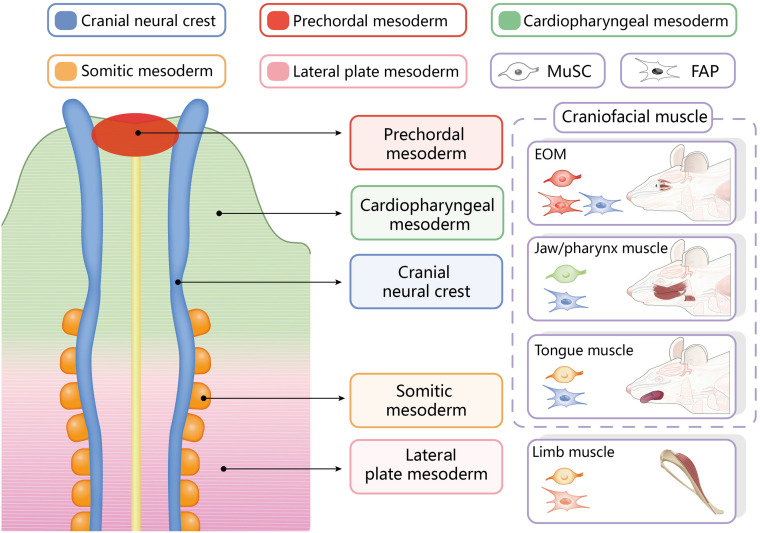
Embryonic origins of resident stem cells in different craniofacial and trunk/limb muscles. EOM, extraocular muscles.

Muscle satellite cells share the ontogeny of the muscles in which they reside (Dumont et al., 2015b). Trunk/limb MuSCs belong to the *Pax3/Pax7* lineage (Nogueira et al., 2015), while the embryonic origins of craniofacial muscle MuSCs can be classified into the following three categories (Schubert et al., 2019; Vyas et al., 2020): (1) extraocular MuSCs are of the *Mesp1/Pitx2* lineage; (2) MuSCs associated with the pharyngeal arch muscles, which include the jaw muscles, facial expression muscles and pharynx- and larynx-associated muscles, which are of the *Mesp1/Isl1* lineage; and (3) MuSCs associated with the tongue muscles, which are of the *Mesp1/Pax3* lineage (Figure 1). In addition to their different lineages, craniofacial MuSCs have distinct myogenic regulatory routes. During the embryonic myogenesis of trunk/limb muscles, *Pax7* typically marks the stem cell state of MuSCs, and its expression precedes that of the myogenic commitment marker *MyoD* (Nogueira et al., 2015; Chang and Kioussi, 2018). In contrast, the expression of *MyoD* in craniofacial muscles during embryogenesis occurs earlier than that of *Pax7*, indicating that the cranial mesoderm is committed to myogenesis before the emergence of craniofacial MuSCs (Nogueira et al., 2015). The *de novo* expression of *Pax7* in craniofacial MuSCs during embryonic development, which is in contrast to its consistent expression in limb and trunk MuSCs, may be a result of their different evolutionary histories (Nogueira et al., 2015).

### Craniofacial MuSCs Behave Distinctly in Homeostasis Maintenance and Regeneration

Because skeletal muscle regeneration largely resembles embryonic muscle development, a significant disparity exists between adult myogenesis in craniofacial and limb muscle regeneration. Craniofacial MuSCs exhibit distinct behaviors both in a homeostatic state and in response to acute and chronic stimuli (Ono et al., 2010; Keefe et al., 2015; Rodriguez et al., 2020a,b).

#### *Mesp1* Lineage Extraocular Muscle MuSCs

Extraocular muscles have long been considered a special muscle group because they are spared in Duchenne muscular dystrophy (Stuelsatz et al., 2015; Verma et al., 2017). Subsequent *in vivo* and *in vitro* analyses provided various evidence to elucidate the mechanisms underlying this observation. The proportion of MuSCs in adult uninjured EOM is five times higher than that in typical limb muscles (Stuelsatz et al., 2015; Verma et al., 2017). The elevated MuSC proportion even persists in aged individuals, and EOM are accordingly more resistant to aging-related degeneration (Formicola et al., 2014; Stuelsatz et al., 2015). Remarkably, EOM undergo more active myonuclei turnover, with nearly 80% of all myofibers undergoing MuSC fusion in the steady state, which is in sharp contrast to the 20% in limb muscles (Formicola et al., 2014). Further analysis revealed that EOM MuSCs could act as a powerful myo-engine, with strong proliferation, differentiation, and self-renewal capacities (La Rovere et al., 2014; Stuelsatz et al., 2015). The transplantation of EOM MuSCs to the limb leads to more vigorous regeneration in the host muscles than in their limb donor counterparts (Stuelsatz et al., 2015). Mechanistic studies indicated that the higher expression of trophic factors in EOM MuSCs, including brain-derived neurotrophic factors and nerve growth factors, may account for the enhanced myogenic activities (Carrero-Rojas et al., 2020).

#### *Mesp1/Isl1* Pharyngeal Arch MuSCs

Pharyngeal arch muscles, also called branchiomeric muscles, constitute the vast majority of craniofacial muscles (Sambasivan et al., 2009; Randolph and Pavlath, 2015), the most studied of which are the first pharyngeal arch-derived masseter and the posterior pharyngeal arch-derived pharyngeal muscles (Sambasivan et al., 2009; Randolph and Pavlath, 2015; Chan et al., 2016). Again, the masseter and pharynx MuSCs each exhibit features distinct from those of trunk/limb MuSCs (Kim et al., 2020).

Unlike in EOM, the proportion of MuSCs in intact masseters is significantly lower than that in their limb counterparts (Pavlath et al., 1998; Ono et al., 2010; Keefe et al., 2015). During aging, however, the number of MuSCs in the masseter increases twofold, which is in contrast to the common decline in the number of MuSCs in aging limb muscles (Ono et al., 2010; Keefe et al., 2015). In response to acute injury, masseter muscles have relatively less efficient regeneration (Pavlath et al., 1998; Ono et al., 2010). Two weeks after a freezing injury, the limb muscles had recovered to their basal level, while the masseter was still infiltrated by a large amount of fibrous tissue. It took 40 weeks for the masseter muscle to fully recover (Pavlath et al., 1998; Yoshioka et al., 2021). The inferior regenerative capacity in the masseter has been correlated with fewer total and proliferating myoblasts during regeneration (Pavlath et al., 1998; Yoshioka et al., 2021). In fact, masseter MuSCs cultured *in vitro* demonstrated more active proliferation but delayed differentiation than those isolated from limb muscles (Ono et al., 2010). Fewer resident MuSCs along with prolonged proliferation and delayed differentiation may explain the inefficient regeneration of the masseter. Nevertheless, transplantation assays revealed that masseter MuSCs could regenerate the host muscles as efficiently as limb muscle MuSCs (Ono et al., 2010).

In pharynx MuSCs, the most prominent feature is more active myonuclei turnover in the absence of induced injury than limb MuSCs (Randolph et al., 2015). A larger proportion of proliferative progeny may be responsible for the elevated contribution of new myonuclei to the uninjured myofibers (Randolph et al., 2015). Another feature is that MuSCs are required for the maintenance of the myonuclear number and myofiber size in certain pharynx muscles, while limb MuSCs do not contribute to muscle mass maintenance (Randolph and Pavlath, 2015; Randolph et al., 2015). Moreover, MuSCs in the nasopharynx, oropharynx and laryngopharynx respond to chronic injury and aging differently (Randolph et al., 2014). Only laryngopharynx MuSCs are sensitive to antiaging treatments intended to combat muscle atrophy, as evidenced by the increased myofiber size and level of central nuclei, a hallmark of MuSC-mediated muscle regeneration (Randolph et al., 2014). The fact that pharynx MuSCs exhibit region-specific characteristics makes them a more complex stem cell group to study.

Apart from their particular regenerative behavior, branchiomeric MuSCs still retain plasticity in cardiogenesis, which is absent in limb MuSCs (Daughters et al., 2017; Huang et al., 2018; Schubert et al., 2019). The expression of the cardiac-specific gene *Tcf21* is evident in cultured adult branchiomeric MuSC (Harel et al., 2009; Daughters et al., 2017). When exposed to *Bmp4* stimuli, branchiomeric MuSCs exhibited drastically elevated expression levels of the cardiogenesis marker genes *Nkx2.5* and *Tbx20*, which were not observed in limb MuSCs cultured under the same conditions (Harel et al., 2009). Although the possibility of manipulating branchiomeric MuSCs to regenerate the heart is still under investigation, preliminary studies have shown that they could be an alternative source for cardiomyocytes (Daughters et al., 2017; Huang et al., 2018).

#### *Mesp1/Pax3* Tongue MuSCs

Tongue muscle regeneration is needed in patients with conditions such as oral cancer invasion into the floor of the mouth (Camacho-Alonso et al., 2020; Kinoshita et al., 2020). Investigations into the MuSC regeneration capacity of this muscle group are rare. The results from the limited studies available demonstrate that myoblasts obtained from somite-derived muscles can regenerate the tongue muscle (Cobourne et al., 2019; Kletzien et al., 2020). The common *Pax3* lineage of the tongue MuSCs and the limb muscle MuSCs may mean that there are relatively more similarities between these two muscle groups. Further investigation into *Mesp1/Pax3* lineage MuSCs is needed to characterize muscle stem cell behavior in this transitional region.

When compared with the trunk/limb MuSCs, all three lineages of craniofacial MuSCs have drastic differences in, homeostasis maintenance and injury response (Table 1). Comparisons among the three different craniofacial MuSCs lineages, however, are currently unavailable. Considering the existing heterogeneity in the developmental origin inside the craniofacial MuSC group, further studies to delineate the differences in MuSC behavior among different craniofacial subgroups are needed to develop a comprehensive understanding of craniofacial MuSC biology.

**TABLE 1:** Embryonic origins and resident muscle satellite cells (MuSCs) cell behavior among craniofacial muscles and trunk/limb muscles.

		Trunk/limb muscles	Craniofacial muscles		
			Extraocular muscles	Jaw/pharynx muscles	Tongue muscles
**Developmental origins**	Myofiber	Somitic mesoderm (Diogo et al., 2015; Sefton and Kardon, 2019)	Prechordal mesoderm (Harel et al., 2009; Vyas et al., 2020)	Cardiopharyngeal mesoderm (Diogo et al., 2015; Vyas et al., 2020)	Anterior somite mesoderm (Diogo et al., 2015; Schubert et al., 2019)
	Connective tissue	Lateral plate mesoderm (Heude et al., 2018)	Neural crest/prechordal mesoderm (Comai et al., 2020)	Neural crest (Heude et al., 2018; Adachi et al., 2020)	Neural crest (Heude et al., 2018; Adachi et al., 2020)
**MuSCs behavior**	Resistance to aging	No	Yes	Masseter: yes Pharynx muscle: no	Unknown
	Engraftment efficiency	Low	High	Masseter: low; Pharynx muscle: unknown	Unknown
	*In vitro* myogenesis	Low	High	Masseter: prolonged proliferation and delayed differentiation; pharynx muscle: unknown	Unknown
	Cardiogenic capacity	No	Unknown	Masseter: yes; Pharynx muscle: no	Unknown

## A Critical Support From the Interstitial Space: FAPs

Fibro-adipogenic progenitors are muscle stromal cells adjacent to the abluminal side of capillaries between myofibers (Lemos and Duffield, 2018). They express the mesenchymal stem cell markers *Sca-1* and *Pdgfr*α (Wosczyna and Rando, 2018). Acting as structural support and signal guides, FAPs are important participants in the regeneration process. They serve as the progenitors of the muscle connective tissue (Vyas et al., 2020) and are the closest anatomical and functional partners of the myofiber (Sefton and Kardon, 2019). When an injury occurs, FAPs infiltrate the damaged area and synthesize extracellular matrix, which provides a framework for myofiber reconstruction (Biferali et al., 2019). Then FAPs undergo programed cell death to avoid excessive fibrogenesis (Lemos and Duffield, 2018; Wosczyna and Rando, 2018; Biferali et al., 2019). Meanwhile, normal FAPs function is indispensable to MuSCs differentiation because it provides promyogenic factors such as *Wisp1* and *Il-10* in a paracrine manner (Biferali et al., 2019). The temporal dynamics of FAPs and MuSCs during muscle regeneration overlap substantially, indicating close intercellular communication (Wosczyna and Rando, 2018).

### Heterogenic and Dynamic Embryonic Origin of Craniofacial FAPs

From an embryogenesis perspective, FAPs share the same origin as muscle connective tissues (Lemos et al., 2012). Generally, the connective tissue within craniofacial muscle is derivative of cranial neural crest, while those within the limb and trunk muscle are lateral plate mesoderm-derived somite (Schubert et al., 2019; Sefton and Kardon, 2019; Vyas et al., 2020). Neural crest cells exert a transient promoting effect on limb muscle myogenesis, but their regulatory role in head muscle development is extensive and lasting. Although it is not necessary for the initiation of craniofacial muscle myogenesis, the cranial neural crest play a crucial role in regulating the migration, patterning and differentiation of craniofacial myogenic precursors (Rinon et al., 2007; Heude et al., 2010). Disruption of this cellular interaction may lead to developmental anomalies, as evidenced in the muscle connective tissue defect identified in patients with hemifacial microsomia (Heude et al., 2010, 2011). Craniofacial muscle patterning is regulated by the surrounding skeletogenic mesenchymal cells derived from the cranial neural crest, while patterning in the trunk/limb muscles is dependent upon signals from the lateral plate mesoderm (Rinon et al., 2007). Strikingly, Wnt signaling acted as an agonist in trunk/limb muscle myogenesis but as an antagonist in craniofacial muscle myogenesis (Rinon et al., 2007).

One distinct characteristic of craniofacial muscle connective tissue is its heterogeneity in embryonic origin (Adachi et al., 2020). Among the seven EOM, the connective tissue associated with four recti muscles is derived from mesoderm, while that associated with the rest three muscles is of neural crest origin (Comai et al., 2020). Furthermore, the composition of craniofacial FAPs undergoes temporal changes after birth (Lemos et al., 2012). Lineage tracing studies revealed that neonatal masseter muscle has the exclusive source of neural crest-derived FAPs, but the mesoderm-derived FAPs blended during subsequent development and growth (Lemos et al., 2012). The proportion of neural crest-derived FAPs in mouse masseter declined to 70% at 5 weeks of age and to 20–30% at adulthood (Lemos et al., 2012). The fluctuation in the composition of different developmental origins during postnatal growth marks another significant characteristic of craniofacial muscle FAPs.

### Distinct FAPs Behavior in Craniofacial Muscle Homeostasis and Regeneration

Although less well studied, FAPs may play relatively more profound roles in craniofacial muscle regeneration. The impetus to study the functional role of FAPs in muscle regeneration only began several years ago, and investigations focusing on craniofacial FAPs are rare. Nevertheless, the putatively disparate performance of neural crest cell-derived craniofacial FAPs has been suggested (Lemos et al., 2012; Formicola et al., 2014; Paylor et al., 2014; Stuelsatz et al., 2014; Comai et al., 2020). Take the EOM muscles, for example, the proportion of FAPs in the MuSC niche in the EOM is much higher than that in the limb, and this trend is maintained in aged individuals and in dystrophic muscles (Formicola et al., 2014). The elevated FAP/MuSC ratio may contribute to the exemption of EOMs from muscular dystrophy-related pathological changes (Formicola et al., 2014). In the notexin-induced masseter regeneration model, neural crest-derived FAPs, rather than mesoderm-derived FAPs, were the main FAP population responsible for muscle regeneration (Paylor et al., 2014).

The ectoderm origin of FAPs has been correlated with a stronger fibrogenesis tendency in craniofacial muscles (Rosero Salazar et al., 2020), which constitutes the pathophysiological foundation of the impaired craniofacial muscle regeneration. Because tissue-specific resident mesenchymal stem cells emerge as targets of fibrosis therapies, as is the case in kidney, lung and skin, FAPs may play a critical role in ameliorating craniofacial muscle fibrosis (Lemos and Duffield, 2018). Because FAPs play pleiotropic roles in inducing fibrogenesis and promoting MuSC differentiation, targeting FAPs alone may suffice to combat fibrosis and enhance myogenesis at the same time.

## Other Myogenic Progenitor Cells in the Stroma

In addition to the above mentioned two types of stem cells, there are other myogenic progenitor cells residing in the interstitial space of muscle. The most studied categories include *Pw1*+ interstitial cells and *Twist2*+ cells.

In the attempt to identify specific stem cells responsible for muscle regeneration, *Pw1*+ cells have garnered a great deal of attention. In fact, the cell stress mediator *Pw1* marks two stem cell populations residing in skeletal muscle: *Pw1*+ */Pax7*+ MuSCs and *Pw1*+ */Pax7-* interstitial cells (PICs). PICs are bipotent progenitor cells that give rise to both smooth muscle and skeletal muscle *in vitro*. Although PICs are not of the *Pax7* lineage, they require *Pax7* for myogenic specification. In addition, a subgroup of PICs share overlapping surface markers with FAPs (*Sca-1* and *Pdgfr*α) and possess fibrogenic and adipogenic capacity, while the *Pdgfr*α- PICs are myogenic (Mitchell et al., 2010; Pannerec et al., 2013).

Another type of interstitial stem cell with myogenic potential is the *Twist2*+ cell. Unlike MuSCs, which contribute to the formation of all types of myofibers, *Twist2*+ cells are type IIb/x fiber specific myogenic progenitors (Liu et al., 2017). Remarkably, the *Twist2+* cells do not express the MuSCs marker *Pax7* but can initiate myogenesis both *in vitro* and *in vivo* (Liu et al., 2017). Once committed to myogenic lineage, these cells downregulated *Twist2* expression and began to express *Pax7* (Liu et al., 2017). A notable difference between MuSC and *Twsit2*+ cells is their opposite roles in muscle regeneration and myofiber size maintenance. MuSCs exert minimal effects on muscle fiber size, as evidenced by the unaffected sarcopenia in *Pax7*-ablated mice (McCarthy et al., 2011; Fry et al., 2015). In contrast, *Twist2*+ cells are required for type IIb/x fiber size maintenance, but their ablation does not affect muscle regeneration (Liu et al., 2017). The hypothesis is that *Twist2*+ cells undergo a pre-myogenic state but are insufficient to guide full regeneration (Liu et al., 2017). It is interesting that *Twist2*+ cells are specifically excluded from the tongue musculature (Liu et al., 2017). Since type IIb/x fibers are the most abundant in all skeletal muscles and are the most susceptible to aging and disease in mice (Liu et al., 2017), further investigation is needed to characterize the myogenesis mechanism in type IIb/x fibers from different muscle lineages to facilitate the treatment of muscle diseases.

## Summary

In contrast to trunk/limb muscles, which are ancestral muscles essential to the support and locomotion of the entire body, craniofacial muscles have long been regarded as a variation on the general body muscle scheme. This variation is manifested in many aspects, including developmental origin, myogenic regulatory trajectory, susceptibility to muscular diseases and regenerative capacity. This discrepancy between craniofacial and trunk/limb muscles may to related to their different embryonic origins and to the Hox gene, which conveys positional memory (Leucht et al., 2008). Studies comparing the bone regeneration process of neural crest-derived mandible and mesoderm-derived tibia revealed that heterogeneity in both the embryonic origin and Hox code could account for the ectopic chondrogenesis instead of osteogenesis in tibia-to-mandible periosteum stem cell transplantation assays (Leucht et al., 2008). A similar genetic marker bearing their ancestor’s phenotypic identity, perhaps HoxA and/or HoxB (Rinon et al., 2007; Vieux-Rochas et al., 2013), might also explain the trunk/limb vs. craniofacial muscle differences. Nevertheless, the mechanism underlying the different regenerative response between craniofacial and limb/trunk muscles may vary, because histological differences already exist, in contrast to the histological equivalency with cellular and molecular disparity in the comparison between mandible and tibia (Mitchell et al., 2010). The concept of positional memory should at least be taken into account. Currently, most observations of the features of craniofacial muscles remain at the level of tissue phenotype or cellular behavior. Investigation into the differences in molecular regulatory mechanisms between craniofacial muscles and trunk/limb muscles or among different subgroups of craniofacial muscles, is needed to provide targets for effective muscle regeneration modalities.

## Author Contributions

XC and JL conceived the idea of writing this manuscript. XC drafted the manuscript. JL drew the figure and critically revised the manuscript. BS critically revised the manuscript. XC and BS provided the funding support. All authors contributed to the article and approved the submitted version.

## Conflict of Interest

The authors declare that the research was conducted in the absence of any commercial or financial relationships that could be construed as a potential conflict of interest.
